# Pollutants Source Control and Health Effects

**DOI:** 10.1155/2013/209739

**Published:** 2013-10-31

**Authors:** Roya Kelishadi, Mohammad Mehdi Amin, Ali Akbar Haghdoost, Ajay K. Gupta, Tuula Anneli Tuhkanen

**Affiliations:** ^1^Pediatrics Department, Child Growth and Development Research Center, Isfahan University of Medical Sciences, Isfahan 81676-3695, Iran; ^2^Environmental Engineering Department, Environment Research Center, Isfahan University of Medical Sciences, Isfahan, Iran; ^3^Research Center for Health Services Management, Institute of Future Studies in Health, Kerman University of Medical Sciences, Kerman, Iran; ^4^International Centre for Circulatory Health, National Heart and Lung Institute, Imperial College, London, UK; ^5^Institute of Environmental Engineering and Biotechnology, Tampere University of Technology, Finland

In the current special issue, different aspects of controlling pollutants and their health effects are being discussed. 

Considering the vicious cycle of rapid urbanization and increasing levels of air pollution, public health and regulatory policies for environmental protection and controlling pollutants from their sources should be integrated into the main priorities of the primary health care system and into the educational curriculum of health professionals [1, 2].

Discovery of the environmental exposures is of crucial importance. It may contribute to or protect against injuries, illnesses, developmental conditions, disabilities, and deaths and the identification of public health and health care actions to avoid, prepare for, and effectively manage the risks associated with harmful exposures. Control of emissions and effluents into air, water, or soil has several beneficial effects at global level [3]. Environmental pollution influences human's health before conception and continues during pregnancy, childhood, and adolescence to adult life [4–6].

It is suggested that such health hazards may represent the greatest public health challenge humanity has faced till now. Studying the effects of environmental factors on the early stages of chronic diseases can serve for future studies and offer strategies for primary preventive interventions [7].

The current special issue seeks to define source control of pollutants and the articles which are related to interaction between environmental pollutants and their effects on health. Its potential topics include recent developments in source control of pollutants, advances in environmental epidemiology, evaluation of mechanisms for action of environmental exposures, the influence of environmental pollutants on human health, and identification of public health and health care policies and measures for risk management.

*Roya Kelishadi* *Roya Kelishadi* *Mohammad Mehdi Amin* *Mohammad Mehdi Amin* *Ali Akbar Haghdoost* *Ali Akbar Haghdoost* *Ajay K. Gupta* *Ajay K. Gupta* *Tuula Anneli Tuhkanen* *Tuula Anneli Tuhkanen*
